# Use of budget savings from patent expiration of cancer drugs to improve affordability and accessibility

**DOI:** 10.1186/s12913-021-06130-y

**Published:** 2021-02-06

**Authors:** Seung Mi Lee, Heui Jae Kim, David Suh, Kyung-In Joung, Eun Suk Kim, Hee Jung Back, Jun Young Kwon, Man-Jae Park, Dong Churl Suh

**Affiliations:** 1 Daegu Catholic University College of Pharmacy, Gyeongsan-si, Gyeongsangbuk-do South Korea; 2 College of Pharmacy, Chung-Ang University Seoul, 84 Heukseok-ro, Dongjak-gu, Seoul, 06974 South Korea; 3 School of Public Health, University of Michigan, Ann Arbor, MI USA; 4 Oncology Market Access, External Affairs, MSD Korea, Seoul, South Korea

**Keywords:** Patent expiration, Cancer drug, Price, Budget savings, Generics, Biosimilars

## Abstract

**Background:**

The introduction of generics after the loss of patent exclusivity plays a major role in budget savings by significantly decreasing drug prices. The aims of this study were to estimate the budget savings from off-patent cancer drugs in 2020–2024 and to inform decision makers on how these savings could be used to improve the affordability of innovative cancer treatments in South Korea.

**Methods:**

A model was developed to calculate budget savings from off-patent cancer drug use in Korea over 5 years (2020–2024). Cancer drugs with one or more valid patents that expire between 2020 and 2024 in Korea were selected. Key input parameters in the model included market share of generics, market growth, and prices of originators and generics. To reflect market dynamics after patent expiration, the trends of the off-patent market were estimated using historical sales volume data of IQVIA from 2012 to 2018. The study assumed that the prices of off-patent drugs decreased according to the price regulations set by the Korean government and that the off-patent market sales volume did not grow. Sensitivity analyses were performed to investigate the uncertainty in model input parameters.

**Results:**

A total of 24 cancer drugs which met selection criteria were identified. In the base case analysis, patent expiration of cancer drugs between 2020 and 2024 could lead to a spending reduction of ₩234,429 million ($203 million), which was 20% of the cancer drug expenditure in the 5-year period. The savings ranged from ₩157,633 million ($136 million) to ₩434,523 million ($376 million) depending on the scenarios in sensitivity analyses.

**Conclusions:**

The findings indicate that patent loss of cancer drugs could lead to a 20% reduction in spending on cancer drugs over the next 5 years in South Korea. The savings could be used to improve the affordability of innovative, advanced cancer drugs for 94,000 cancer patients by reallocating the budget savings from patent expiration.

## Background

The estimated global incidence of cancer was 18.1 million cases with 9.6 million deaths in 2018. The most commonly diagnosed cancer was lung cancer, accounting for 11.6% of the incidence, closely followed closely by breast cancer (11.6%) and prostate cancer (7.1%) [1]. Oncology drugs account for the largest segment in any specialty medical area with rapidly growing expenditures, which have been recognized as a major worldwide concern. Global expenditures on cancer drugs ranged from US$74 billion to $84 billion in 2017 and project to reach between $220 billion and $250 billion by 2023 due to an aging population and the development of new yet costly treatment methods [2, 3]. Cancer incidence in South Korea was 232,000 with 78,000 deaths in 2017. The most commonly diagnosed cancer was stomach (12.8%), followed by colorectal (12.1%), lung (11.6%), thyroid (11.3%), breast (9.6%), and liver (6.6%) [4]. Cancer drug expenditures for these six major cancers accounted for 37% of the US$2658 million expenditures in 2015, and the annual cancer drug cost per person was US$2149 [5].

Adequate budget allocation for limited health resources is needed to alleviate the price burden to improve access to innovative drugs, and various types of cost-containment policies, such as generic/biosimilar drug substitution, actual transaction pricing, price-volume agreements, and patient access schemes, are used in many countries [6]. The introduction of generics/biosimilars after the loss of patent exclusivity plays a major role in budget savings by significantly decreasing drug prices. Drug prices decreased in the time period 1–5 years after patent expiration, with the magnitude ranging from 6.6 to 66% of the price at the time of expiration, as evidenced in Australia, Canada, the United States, and Europe [7]. In recent years, the extent of the price reduction of originators and generics/biosimilars has greatly increased. Generics/biosimilars that entered the market between 2011 and 2013 underwent a 79% price reduction within 1 year of patent expiration compared with the 44% price reduction for generics/biosimilars that entered the market between 2002 and 2004 in the United States, and their prices continued to fall in subsequent years [8]. The loss of exclusivity of originators provides significant savings for patients, healthcare payers, and the government and thus increases the affordability and coverage of innovative drugs.

South Korea has used the positive list system since 2007 to allocate health resources more effectively for new drugs. Under the positive list system, only cost-effective drugs are reimbursed based on the submission of prices and reimbursement applications from pharmaceutical companies on a voluntary basis. However, the listing of costly drugs, especially cancer drugs, is challenging due to uncertainties in financial burden and cost-effectiveness. From 2007 to 2014, 16 (51.6%) out of 31 evaluated cancer drugs were accepted for reimbursement [9]. To improve patient access to new advanced drugs, the Korean government has implemented a variety of policies including the risk-sharing scheme, essential drug designations, and exemption from submitting the cost-effectiveness analysis results for new drugs. The risk-sharing scheme in Korea has increased the approval of costly cancer drugs for reimbursement from 13 to 47% and contributed to improve patient access without additional financial burden on the government [10, 11].

In July 2020, the Korean government introduced a new stepwise pricing policy for the off-patent market to decrease the price of off-patented market drugs depending on two criteria, i.e., whether or not the bioequivalence test was performed by the generic company without a consigned external vendor, whether the ingredient used in the production process of generics was certified by the MFDS [12]. Depending on whether the generics met one or more of the aforementioned criteria, the reduction in price varied as follows: the price would be reduced to approximately 38.69% (i.e., if generic does not meet any of the two criteria), 45.52% (i.e., meet one of the criteria), or 53.55% (i.e., meet two of the criteria) of the originator’s price for up to the first 20 generics after patent expiration. For subsequent generics, the price would be 85% of the existing cheapest generics. This policy would further increases sustainability, affordability, and access to innovative yet costly drugs.

By regulating drug prices, significant budget savings after originator patent expiration can be projected in the future; however, no study has been conducted on the projected budget impact after the patent expiration of cancer drugs. The objectives of this study were to estimate the budget savings according to different scenarios for cancer drugs that would lose their patents between 2020 and 2024 and to inform decision makers on how the savings could be reallocated to address the affordability concerns facing patients who require access to innovative treatment options, which would eventually improve overall health outcomes in South Korea.

## Methods

### Model structure

A model was developed to calculate budget savings from the patent expiration of cancer drugs in a 5-year period from 2020 to 2024. The model was operationalized with market dynamic variables including the market share of generics/biosimilars, market growth of therapeutic classes in which the originator would lose market exclusivity, the originator’s price level, and the price level of generics/biosimilars after patent expiration. The terms “generics” and “biosimilars” are used interchangeably in this study. The key outcome variables in the model were drug expenditures on a yearly basis due to the introduction of generic/biosimilars into the market and drug expenditures assuming no generic/biosimilar. The budget savings were calculated as the differences between these two amounts.

The model for base case analysis assumed that the market share of generics/biosimilars during the study period (2020–2024) was proxied by the market dynamics of off-patent cancer drugs between 2012 and 2018 (i.e., proxy case analysis). The prices of originators and generics/biosimilars after patent expiration were assumed based on the price regulations set by the Korean Ministry of Health and Welfare in April 2012 [13]. The market size of the off-patent market was assumed to be the same as that of the market at the time of patent expiration as a conservative estimate, as the cancer drug market in Korea is not price-sensitive due to the 5% coinsurance for cancer treatment. Several scenarios were examined in sensitivity analyses by varying model parameters such as inclusion of different cancer drug groups, delay in generic/biosimilar entrance, the price level of originators and generics/biosimilars, adjustment for proportion of reimbursement, the growth of the off-patent cancer drug market, the risk sharing scheme rebate, and the new generic pricing policy across a range of relevant values.

The annual sales of cancer drugs with patents that would expire between 2020 and 2024 were predicted using the quarterly IQVIA data from 2012 through 2018. The model was developed and analyzed using Microsoft Excel. The sales and budget amounts were presented in Korean Won (₩) currency and were converted to the United States Dollars ($) using a currency exchange rate of ₩1 = $0.000865052 as of 31 December 2019.

### Data source and drug selection

This study was performed using the quarterly IQVIA data from 2012 to 2018. This study selected cancer drugs classified as L01 (antineoplastic agents), L02 (used in endocrine therapy specifically in the treatment of neoplastic diseases), or L04 (immunosuppressants limited to siltuximab, lenalidomide, and pomalidomide) based on the Anatomical Therapeutic Chemical (ATC) classification system or those with cancer treatment indications [14]. In addition, the selected drugs based on the ATC classification system had to have at least one or more valid patents that would expire between January 2020 and December 2024, as the patent expiration would lead to the introduction of generics/biosimilars to the off-patent market. The patent expiration date of the drugs was retrieved from the product patent information page on the website of the Ministry of Food and Drug Safety’s (MFDS; equivalent to the Food and Drug Administration in the United States) or based on post-market surveillance data [15].

**Fig. 1:**
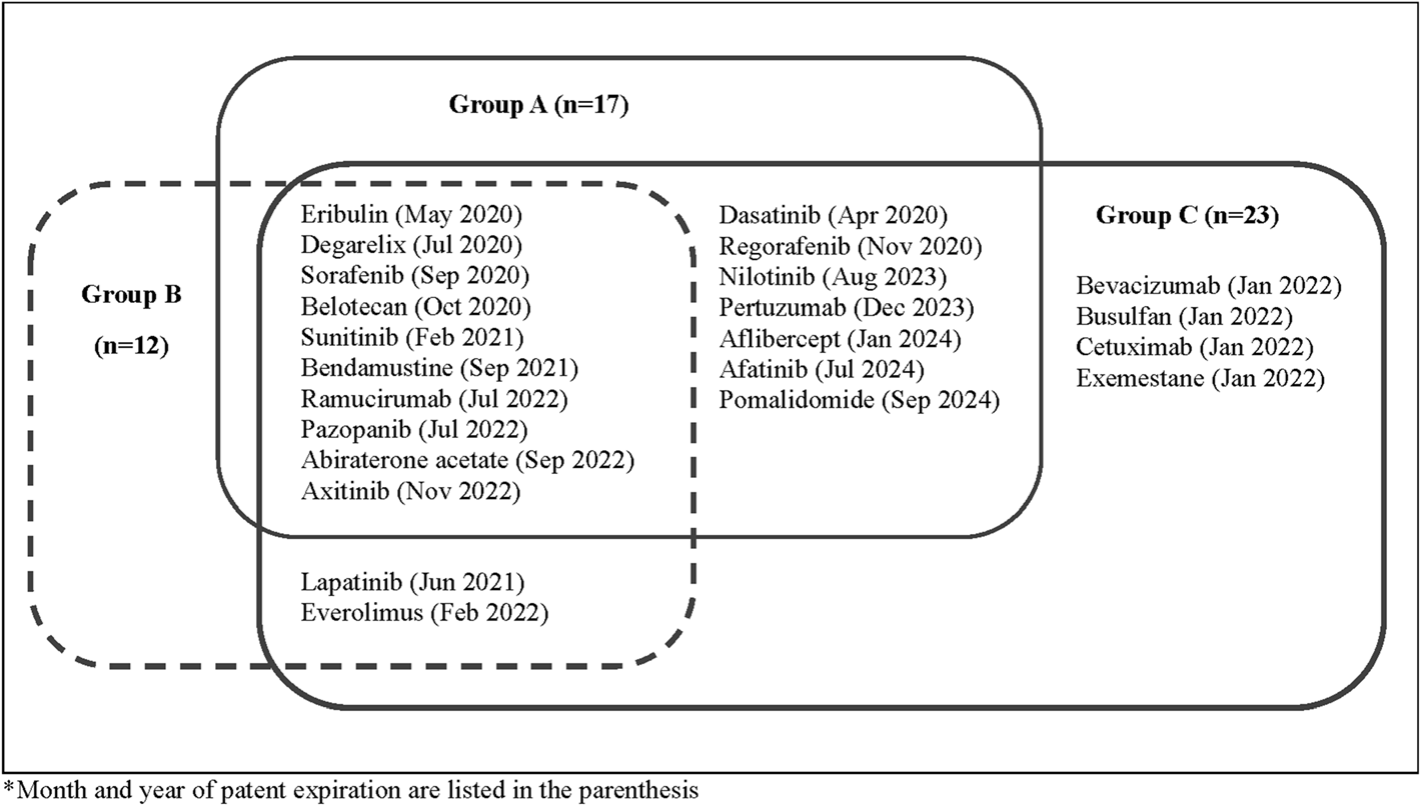
Study cancer drugs included in the analyses

The study drugs were divided into three groups depending on the patent expiration dates as listed in Fig. 1. If more than one patent expiry occurred during the study period, the earliest patent expiration date was selected because the majority of proxy cases have shown that generics/biosimilars enter the market after the expiration of the first valid patent. Group A (i.e., base case group) included 17 cancer drugs with at least one valid patent that would expire during the study period. Group B (i.e., delayed group) included 12 cancer drugs in which at least one patent was expired prior to the beginning of the study period, but later patents would expire during the study period. For the drugs in Group B, the latest date of patent expiration was used as a more conservative approach. Group C (i.e., opportunistic group) included 23 cancer drugs classified in group A and group B and drugs that were off-patent as of 31 December 2019; however, the exact date of patent expiration was unavailable. It was assumed that the generics/biosimilars of these drugs would be launched at any time during the study period; thus, the patent expiration date was proxied as January 2022, as an approximate midpoint of the study period.

### Analysis of proxy cases

An analysis of proxy cases was conducted to estimate dynamic market variables during the study period based on the historical trends of the off-patent market for 2012–2018. Input parameters including market share of generics/biosimilars, market growth of the off-patent market, and price trends of originators and generics/biosimilars were estimated using the historical records of 14 drugs selected based on the inclusion and exclusion criteria below.

The cancer drugs selected for the proxy case analysis were class II cancer drugs for reimbursement classified by the Health Insurance Review & Assessment Service (HIRA) [16]. The HIRA classified all cancer drugs into two classes based on the year of drug development, status of re-evaluation for efficacy and safety, and status of orphan drugs. Class I cancer drugs include traditional cancer drugs with a long history of usage, and Class II cancer drugs include recently developed cancer drugs. Among 91 class II cancer drugs, the drugs under consideration for this study were excluded if they did not have generics/biosimilars after patent expiration (*n* = 63), were not the originator (*n* = 1), were an orphan drug (*n* = 3), had their first generics/biosimilars before 2012 (*n* = 8), did not fit the inclusion criteria of the ATC classification system (*n* = 1), or were registered but not marketed (*n* = 1). After applying the exclusion criteria, 14 cancer drugs were selected including anagrelide, azacitidine, bortezomib, capecitabine, decitabine, erlotinib, gefitinib, imatinib, lenalidomide, pemetrexed, rituximab, temozolomide, topotecan, and trastuzumab. Drugs with their first generics/biosimilars in 2012–2018 were included because the current pricing scheme following patent expiration has been implemented since 2012 [13, 17, 18].

The input parameters for market dynamics after patent expiration are summarized in Table 1. The variables of the proxy market were estimated after the market was stratified by chemical drugs and biologics due to different pricing policies and market dynamics using the IQVIA sales data from 2012 to 2018. Sales volume units were converted to consumption units based on the daily dose divided by the daily maintenance dose approved in Korea to adjust for a variety of dose units for each drug. The market share of generics/biosimilars for each year was projected as a proportion of generics/biosimilars in relation to the wholesale volume unit, and the value of the first year and the average of annual additive market share were calculated for market share parameters. Chemical generics were estimated to account for 5.0% of the market in the first year after patent expiration and increase in the off-patent market by 2.1% annually thereafter. The market share of biosimilars was estimated to be 3.1% of the market in the first year and subsequently increase to 12.5% in the second year. As information after the second year was not available due to the limited sales data of biosimilars for proxy cases, this model conservatively assigned the same market share of 12.5% from the third year onwards. The price levels of the originators and generics/biosimilars after patent expiration were continuously decreased over 5 years.

**Table 1:** Input parameters for market dynamics after patent expiration

Variables	Years after patent expiration				
	1st year	2nd year	3rd year	4th year	5th year
Market share of generics/biosimilar					
Chemical generics	5.0%	7.1%	9.2%	11.3%	13.4%
Biosimilars	3.1%	12.5%	12.5%	12.5%	12.5%
Market growth					
Chemical drugs	1.3%	1.4%	1.4%	1.4%	1.4%
Biologics	4.0%	0.0%	0.0%	0.0%	0.0%
Price level of originators^a^					
Chemical drugs	75%	60%	60%	53%	53%
Biologics	80%	80%	80%	80%	80%
Price level of generics/biosimilars^a^					
Chemical generics	58%	57%	55%	51%	51%
Biosimilars	72%	57%	57%	57%	57%

^a^ For the price level, the percentages at each year after patent expiration are the relative ratio compared with the price (=100%) at the time of patent expiration

The price levels estimated using proxy cases were used for sensitivity analyses. To calculate the actual market price of proxy cases, the price cap and enforcement date of each drug were obtained from HIRA’s website, which publishes the price of drugs listed in the national health insurance drug formulary [19]. After confirming the timing of the price changes and the price of the proxy cases after patent expiration, the price levels for each year after patent expiration were calculated and compared with the price at the time of patent expiration. Based on the sales volume at the time of patent expiration according to the IQVIA data, the weighted average of price levels was calculated for originators and generics/biosimilars for each year.

### Sensitivity analyses

One-way sensitivity analyses were performed to measure the relative impact of key input parameters on the robustness of the base case budget savings. Sensitivity analyses were performed for different groups of cancer drugs: group B cancer drugs were included to estimate the impact of longer patent protection, and group C cancer drugs were included with the assumption that off-patent cancer drugs would have generics/biosimilars in 2022 to capture the potential additive impact. Sensitivity analyses were also performed with the adjustment of the IQVIA sales volume to reflect the reimbursed amounts for approved indications based on the total sales. As the HIRA does not reimburse for the use of unapproved indications, 90% of the IQVIA sales volume was assumed to be reimbursed. Uncertainty associated with the price levels of originators was investigated using the price trends of proxy cases to reflect actual market dynamics.

This study also assessed the impact of a 6-month delay of generic/biosimilar entrance into the market and stability of budget savings to changes in market growth. Market growth after patent expiration was estimated at 1.3% in the first year and 1.4% thereafter for chemical drugs based on the trends of the off-patent market determined by proxy case analysis to reflect market dynamics. For biologics, market growth after patent expiration was 4.0% in the first year and remained the same thereafter due to the lack of biosimilar sales data for proxy cases. In addition, the impact of the confidential rebate magnitude of originators, which contracted with the risk-sharing scheme, on the robustness of budget savings was analyzed by assuming a 25% rebate [20].

Sensitivity analysis was also performed for a new stepwise pricing policy, enacted on July, 2020, which would decrease the price of drugs in the off-patented market. The price of generics/biosimilars would be determined depending on the bioequivalence test by the generic company, the quality of raw materials used for generics, and the number of generics in the market [12].

## Results

**Fig. 2:**
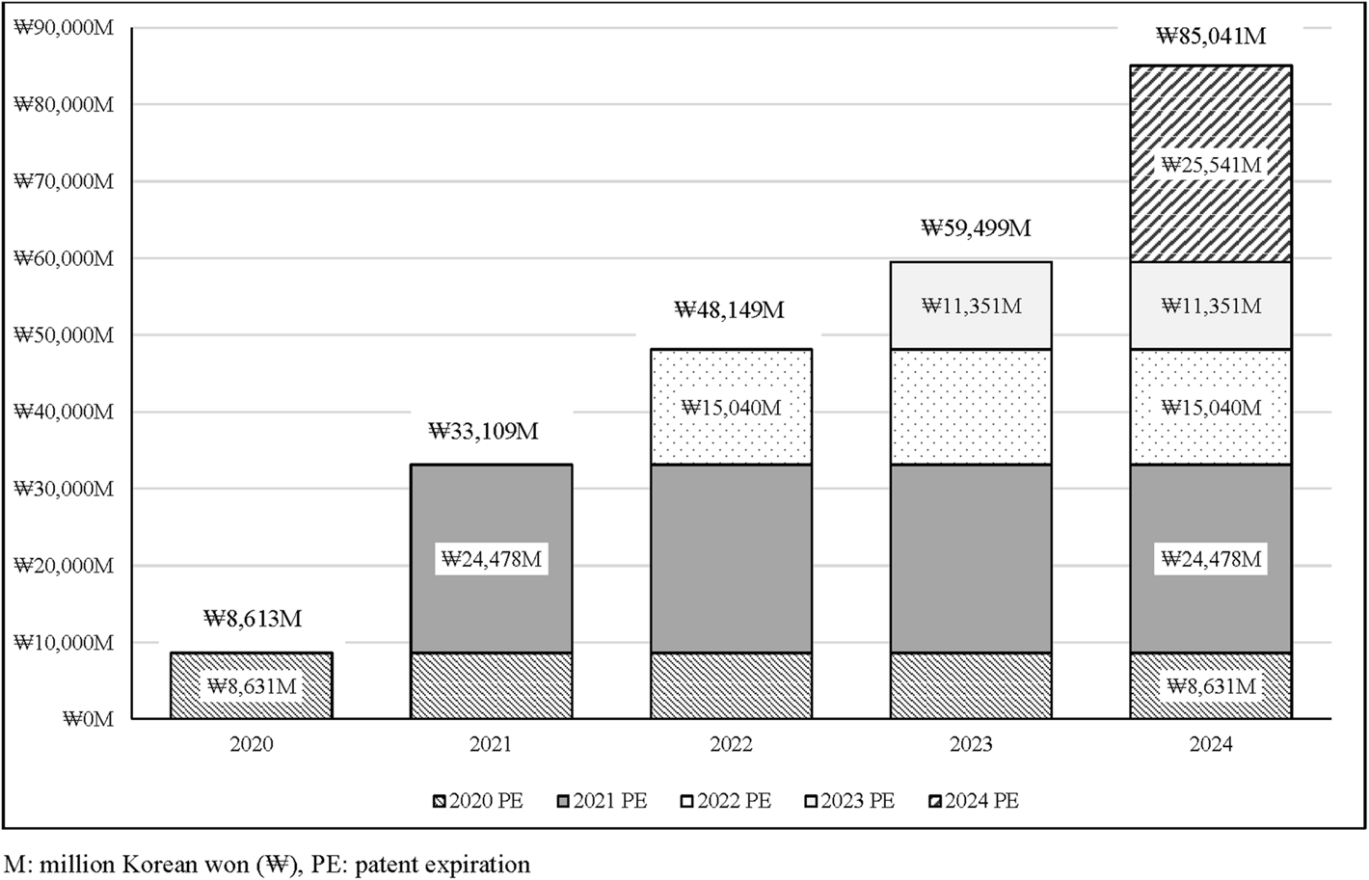
Cumulative budget savings according to the year of patent expiration during 2020–2024

Table 2 shows the potential budget savings in the base case analysis of 17 originators (group A), which would lose market exclusivity between 2020 and 2024. The annual drug expenditures of the study originators prior to patent expiration were estimated at ₩229,094 million ($198 million) in 2019 and are expected to be decreased to ₩144,054 million ($125 million). The total savings from patent expiration over the next 5 years were ₩234,429 million ($203 million), which were equivalent to 20% of the 2019 drug expenditure. The annual savings continuously increased during the study period. Figure 2 shows the cumulative annual savings of a given year based on prior year’s savings. The total budget savings of ₩85,041 million in 2024 were the sum of the savings of ₩8631 million ($7 million; 10.1%) in 2020, ₩24,478 million ($21 million; 28.8%) in 2021, ₩15,040 million ($13 million; 17.7%) in 2022, ₩11,351 million ($10 million; 13.3%) in 2023, and ₩25,541 million ($22 million; 30.0%) in 2024.

**Table 2:** Annual drug expenditures and budget savings from patent expiration during 2020–2024

Year	Expected drug expenditures		Budget savings^a^		
	Korea (₩): million	US ($): million	Korea (₩): million	US ($): million	(% of 2019 expenditure)
2019	₩229,094	$198			
2020	₩220,464	$191	₩8631	$7	4%
2021	₩196,173	$170	₩33,109	$29	14%
2022	₩180,946	$157	₩48,149	$42	21%
2023	₩169,595	$147	₩59,499	$51	26%
2024	₩144,054	$125	₩85,041	$74	37%
Total 2020–2024	₩911,232	$788	₩234,429	$203	20%

The Korean won (₩) was converted to the United States Dollar ($) using a currency exchange rate of ₩1 = $0.000865052 as of 31 December 2019

^a^ Budget savings were calculated by subtracting the expected expenditure in a given year from the drug expenditure in 2019

**Fig. 3:**
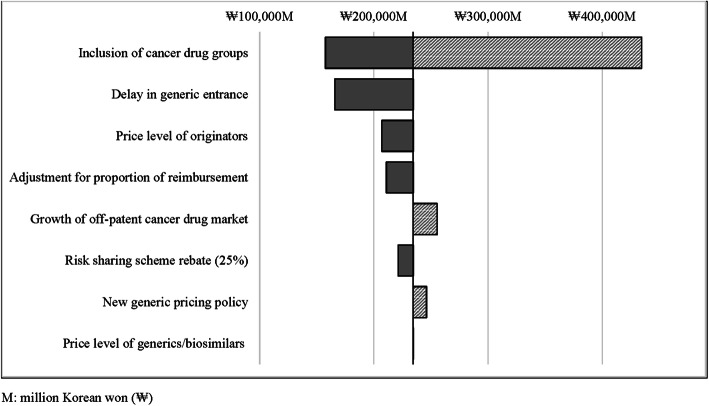
Sensitivity analyses for estimating the impact of input parameters on budget savings during 2020–2024

The results from one-way sensitivity analyses, which evaluated the impact of changes in input parameters, are shown in Fig. 3. The results showed that uncertainty in savings was mainly driven by the changes in the list of drugs depending on the drug group, followed by the order of timing of generic entrance (i.e., delay in generic entrance), price level of originators, adjustment for proportion of reimbursement, the growth of the off-patent cancer drug market, and manufacturers’ rebate percentage in the risk-sharing agreement. Table 3 revealed budget savings for different scenario analyses. When the scenario included drugs that were off-patent or lacked patent information (i.e., Group C cancer drug), in addition to drugs in the base case group, the budget savings for 5 years could be increased up to ₩434,523 million ($376 million), which was almost double the amount of the base-case savings. However, the more conservative approach based on a later off-patent date led to a decreased magnitude of savings of ₩157,633 million ($136 million) which was 67% of the base-case savings amount. The delayed entrance of generics reduced budget savings by 29%, which provided savings of ₩165,904 million ($144 million). If the off-patent market is assumed to grow at the growth rate obtained from the proxy case analysis, the savings would increase to ₩255,316 million ($221 million), which is a 9% increases from the base-case scenario. When the new stepwise pricing policy was applied to the model, the expected total savings increased by 5%, which resulted in savings of ₩246,082 ($213 million).

**Table 3:** Total savings from patent expiration during 2020–2024 for various scenarios

Scenario	Budget savings			Changes from base case savings		
	Korea (₩): million	US ($): million	% of expenditures^a^	Korea (₩): million	US ($): million	(%)
Base case	₩234,429	$203	20%			
Based on group B cancer drugs	₩157,633	$136	14%	- ₩76,795	-$66	−33%
Based on group C cancer drugs	₩434,523	$376	38%	₩200,095	$173	+ 85%
Generic entrance: 6 months delay	₩165,904	$144	14%	- ₩68,524	-$59	−29%
Risk-sharing scheme rebate of 25%	₩221,465	$192	19%	-₩12,963	-$11	−6%
Adjustment for reimbursement	₩210,985	$183	18%	- ₩23,443	-$20	−10%
Growth of off-patent market	₩255,316	$221	22%	₩20,888	$18	+ 9%
Price level of originators	₩206,929	$179	18%	-₩27,499	-$24	−12%
Price levels of generics/biosimilars	₩234,771	$203	20%	₩343	$0	0%
New pricing policy (July 2020)	₩246,082	$213	21%	₩11,654	$10	+ 5%

^a^ The proportion of budget savings was calculated by dividing budget savings for each scenario by the 5-year total expenditure for the base case (₩1,145,470 million)

## Discussion

This study projected the potential budget savings from off-patent cancer drug prices and the launch of generics/biosimilars in a 5-year period. The patent expiration of cancer drugs leads to a total budget savings of ₩234,429 million ($203 million), with a range from ₩157,633 million ($136 million) to ₩434,523 million ($376 million) under different scenarios, which was 20% of the cancer drug expenditure in 2020–2024 in South Korea. An additional of 94,000 cancer patients could be treated in South Korea over five years by reallocating the ₩234,429 million ($203 million) savings. The savings are estimated to be smaller than those of other countries, such as members of the EU or the United States, because generic substitution by pharmacists after patent expiration in South Korea is extremely rare (less than 1%) and competition in the off-patent market is not severe due to fixed price regulation for the off-patent market in Korea.

After the listing of new drugs in the national formulary, drug prices are strictly monitored by the Korean government through actual transaction pricing and price-volume agreements. Under an actual transaction price monitoring system, the price is adjusted to the level of the weighted average actual transaction price in the hospital and pharmacy sectors. In the price-volume agreement scheme, the price adjustment is negotiated with the national health insurance service if the sales value is higher than 30% of the agreed upon volume in the first year, and the maximum price is adjusted by 10%. The average price reductions from actual transaction pricing and price-volume agreements range from 0.94 to 6.6% [13]. Prices are further reduced if the indications of a drug are expanded for potential use.

The Korean government implemented a risk sharing agreement in December 2013 for cancer or orphan drugs without alternatives or for drugs used to treat life-threatening diseases to expedite patient access. In June 2015, a policy for exempting cost-effectiveness assessment was implemented for drugs without alternatives; intended for indications for life-threatening diseases; approved based on the results of randomized clinical trials with a single arm; or for those with difficulty in obtaining sufficient evidence due to targeting rare diseases [21]. After the implementation of this policy, the rate of reimbursement approval was slightly increased from 47 to 50% for high-cost cancer drugs [10].

Despite these efforts, the adoption rate for new cancer drugs (51.6%) was still lower than that for new drugs in other therapeutic areas (71.6%) [9]. Access to innovative drugs is still limited due to approval for restricted indications or lag time between approval and reimbursement. For instance, reimbursement for anti-PD-1/PD-L1 is currently restricted for narrow indications despite its proven clinical and economic benefits. Policies to improve patient access to innovative cancer drugs can achieve optional outcomes when they are implemented in conjunction with the reallocation of limited resources. Budget savings from patent expiration could be used to increase access to innovative therapy for patients with cancer.

The price impact of patent expiration varies among different countries. The reduced price of originators and entry of generics/biosimilars can lead to significant savings for patients and the government. In the United States, generics/biosimilars accounted for 90% of the prescriptions dispensed in 2018, an increase from 75% in 2009 [22]. The amount of savings from patent expiration and the availability of generics//biosimilars was expected to increase by 47% from $72 billion in 2014–2018 to $106 billion in 2019–2023. In the case of atorvastatin, the introduction of generics/biosimilars led to budget savings of 28% in atorvastatin expenditures in 2012–2014 despite a 20% increase in overall atorvastatin use in the United States [23]. Similarly, the amount of savings from the loss of patent exclusivity for tyrosine kinase inhibitors in the United States over the next 5 years has been projected to be 39% (cumulative savings = $12.2 billion) [24]. After the introduction of atorvastatin generics, a limited number of studies were conducted and published on changes in market expansion, the level of price cuts, and factors contributing to generic prescriptions but these studies did not estimate nationwide savings from the introduction of generics in Korea [25, 26].

Cost-saving strategies can promote value-based patient care. The prices of biosimilars may vary in different countries depending on the country’s regulations regarding healthcare expenditures. The prices of biosimilars have been found to be 10–30% lower than those of original biologics [27–30]. Further reduction in generic prices could help policymakers in making evidence-based decisions to reallocate resources for costly novel drugs or coverage expansion. A recent study has indicated the benefits of off-patent biologics and biosimilars [29]. Savings from patent expiration could be used for the reimbursement of new advanced drugs, to initiate treatment with advanced drugs, to increase patient accessibility and affordability of innovative treatments, or to increase the number of healthcare providers. In addition, market competition between off-patent biologics and biosimilars might lead to incremental innovation.

The accessibility of high-cost cancer drugs can be improved by efficient budget allocation. Possible strategies to improve the affordability of cancer drugs include value-based price negotiation by the government, sufficient availability of quality-assured generics/biosimilars and biosimilars, and patent assistance programs from pharmaceutical companies [31]. Countries that adopted the health technology assessment approach, such as Australia, Canada, France, and the United Kingdom, have demonstrated flexible willingness-to-pay thresholds for an incremental cost-effectiveness ratio depending on the disease severity, thus providing patient access to costlier and rare oncologic drugs that would not be otherwise available based on traditional measures of cost effectiveness [32, 33]. These health policies can be considered by the Korean government to improve the affordability of more innovative drugs.

Policymakers need to address the strategies used by originators to delay the approval of generic drugs and to create systems to minimize the delay in generic market saturation after patent expiration [34]. The Hatch-Waxman Act in the US encourages the rapid entry of generics/biosimilars using an Abbreviated New Drug Application after patent expiration; however, there are still barriers to market entry for generics/biosimilars due to a 6-month market exclusivity incentive for the first generic after patent expiration. The introduction of low-cost biosimilars could lead to further savings in drug expenditures; thus, delays in market saturation would result in higher costs for individual patients and the health care system.

Most previous studies did not determine the projected savings for a range of cancer drugs; instead, they reported substantial savings from using individual generics/biosimilars or biosimilars. To the best of our knowledge, this study is the first to project budget savings based on various cancer drugs scheduled for patent expiration between 2020 and 2024. Budget savings were estimated to reflect real-world situations after patent expiration using proxy case analysis with actual sales data. As the current pricing policy on generics/biosimilars has been in implementation since 2012, the trends of the off-patent cancer drug market over the 5 year study period were proxied by the past trends of generics/biosimilars that lost their exclusivity in 2012–2018 [13, 17]. The findings of this study should be interpreted with an understanding of its limitations. Because this study estimated future financial savings from patent expiration, this study involves uncertainties associated with changes in the current pricing policy, estimation of input parameters using historical sales data, and savings estimation for different scenarios. The base case model did not consider pricing strategies and market dynamics after patent expiration because the prices of most drugs were determined based on a price policy strictly regulated by the Korean government. However, this study performed sensitivity analyses to estimate the impact of dynamic market variables on savings. Patient access to innovative cancer drugs may be improved by the reallocation of resources within the therapeutic treatment of cancer. More importantly, budget savings from patent expiration can be achieved in various therapeutic drug classes.

## Conclusions

This study estimated that the patent expiration of originators would lead to total budget savings in cancer drug expenditures of $203 million (₩234,429 million), which was 20% of cancer drug expenditures in 2020–2024 in South Korea. These budget savings could be used to treat an additional of 94,000 cancer patients. The reallocation of these savings could improve the affordability of innovative and advanced cancer drugs, eventually improving the quality of life of patients with cancer. The actual savings would be higher if the savings were estimated based on various therapeutic classes of drugs that would be off-patent and impact of the new stepwise drug pricing policy from July 2020 were considered. Further studies are recommended to estimate the magnitude of budget impact for all therapeutic areas and to determine mechanisms of allocating limited resources more efficiently in Korea.

## Data Availability

The data that support the findings of this study are available from the IQVIA. However, restrictions apply to the availability of these data, which were used under license for this study. The data are available from the corresponding author upon reasonable request with the permission of the IQVIA. The data used for the drug patent information are available from the public domain: Ministry of Food and Drug Safety (reference [15]).

## Abbreviations

ATC: Anatomical therapeutic chemical
MFDS: Ministry of Food and Drug Safety
HIRA: Health Insurance Review & Assessment Service
